# Analysis of multi-site HPV infection and vaccination willingness among men who have sex with men in Tianjin, China

**DOI:** 10.3389/fpubh.2024.1453024

**Published:** 2024-10-14

**Authors:** Jianyun Bai, Xiaoyue Dong, Tielin Ning, Jingjin Zhu, Ziming Wu, Huijuan Li, Maohe Yu

**Affiliations:** ^1^Tianjin Centers for Disease Control and Prevention, Tianjin, China; ^2^Tianjin Key Laboratory of Pathogenic Microbiology of Infectious Disease, Tianjin Centers for Disease Control and Prevention, Tianjin, China; ^3^School of Public Health, Tianjin Medical University, Tianjin, China

**Keywords:** human papillomavirus (HPV), HPV vaccines, human immunodeficiency virus (HIV), men who have sex with men (MSM), influencing factors, vaccine willingness

## Abstract

**Background:**

Men who have sex with men (MSM) are vulnerable to HPV infection. This study aims to explore the HPV infection status at different sites among HIV-positive MSM, HIV-negative MSM, and men who have sex with women (MSW), and to investigate their willingness to receive HPV vaccination.

**Methods:**

From September 2023 to April 2024, three groups were recruited in Tianjin, China. Participants completed an electronic self-administered questionnaire, which included demographic information, knowledge related to sexually transmitted diseases, behavioral information, and willingness to receive the HPV vaccine. Samples were collected from the anal region, genitals, and oral cavity for HPV typing.

**Results:**

A total of 1,559 participants were recruited, including 300 HIV-positive MSM, 600 HIV-negative MSM, and 659 MSW. The HPV infection prevalence for any site were 62.0, 53.7 and 8.3%, respectively (*p* < 0.001). The infection prevalence for HPV genes covered by the 9-valent vaccine were 47.0, 36.8, and 3.5%, respectively (*p* < 0.001). Co-infection prevalence at anal and genital were 20.3, 14.2, 0.6%, respectively. Co-infection prevalence at anal and genital and oral were 1.3, 0.3%, 0, respectively. A total of 77.0% HIV-positive MSM and 75.3% HIV-negative MSM expressed willingness to receive the HPV vaccine, whereas 58.9% of MSW were unwilling (*p* < 0.001). Being HIV-positive (aOR, 3.119; 95% CI, 2.213–4.395), being over 46 years old (aOR, 1.994; 95% CI, 1.266–3.142), with an occupation classified as “white collar workers” (aOR, 1.620; 95% CI, 1.111–2.362) and “freelancing” (aOR, 2.025; 95% CI, 1.371–2.993) and a history of homosexual behavior in the past 6 months (aOR, 5.338; 95% CI, 3.802–7.495) were risk factors for HPV infection among men in Tianjin. Consistently using condoms in the past 6 months (aOR, 0.667; 95% CI, 0.513–0.867) were protective factors.

**Conclusion:**

The HPV infection prevalence among MSM in Tianjin is significantly higher than among MSW, with higher prevalence in the anal region compared to the genital and oral region. HPV infection is associated with HIV infection, older age, and homosexual behavior. Most MSM showed a positive willingness to receive the HPV vaccine, indicating the necessity to implement targeted HPV vaccination programs for MSM and to enhance necessary preventive knowledge and behavioral interventions.

## 1 Introduction

Human papillomavirus (HPV) infection is one of the most common sexually transmitted infections (STIs) (1). Previous studies indicated that HPV infection is a significant contributing factor in the development of cervical cancer (2), anal cancer (3), and genital warts (4).

Men who have sex with men (MSM) are at high risk for HPV infection and transmission due to their unique sexual behavior (5) and multiple and casual sexual partnerships. A meta-analysis published in 2019 revealed that the prevalence of any anal HPV genotype infection was significantly higher among MSM compared to men who have sex with women (MSW), with rates of 79 vs. 43% in HIV-positive men and 47 vs. 12% in HIV-negative men (6). A meta-analysis published in 2021 indicated that the prevalence of anal HPV infection among MSM in China ranged from 53.60 to 85.10%, with a higher prevalence of high-risk HPV types in northern China (7).

HPV vaccination is an effective method for preventing HPV-related diseases in both men and women. An observed vaccine efficacy rate of 60.2% in men (8), and efficacy of the qHPV vaccine against anal intraepithelial neoplasia was 77.5% in the per-protocol efficacy population (9). In mainland China, the 2-valent, 4-valent, and 9-valent HPV vaccines were approved for use (in women) in 2016, 2017, and 2018, respectively (10). However, there has been no specific HPV vaccination program for men. It is of great importance to comprehend the willingness of men to receive the HPV vaccine if future HPV vaccination efforts are to be feasible. Any concerns that men may have about receiving the vaccine need to be addressed. Despite several studies reporting the prevalence of HPV among Chinese MSM, the majority of those studies have focused on the prevalence of HPV infection in anal region (11–13). Few studies investigated the prevalence of multi-site HPV infections among different types of men in a single city. It is estimated that there will be significant differences in HPV infection rates among individuals with different sexual behaviors, so this study examines the prevalence and distribution of HPV genotype at anal, genital, and oral sites among HIV-positive and HIV-negative MSM, and MSW in Tianjin. Additionally, the study examines the willingness of those individuals to receive the HPV vaccine, providing a reference for the development of comprehensive HPV prevention strategies and for the evaluation of the clinical application value of the HPV vaccine in different male populations.

## 2 Materials and methods

### 2.1 Ethical approval

The study was approved by the Ethics Committee of the Tianjin Center for Disease Control and Prevention (Approval no. TJCDC-R-2023-013).

### 2.2 Study population

The calculation of sample size is based on the sample size formula *N* = 400 × (Q/P) of the current situation survey, where P is the HPV infection rate and Q = 1-P. According to previous literature reports (7), the HPV infection rate of HIV positive men who have sex with men is calculated according to *p* = 0.60, and the HPV infection rate of HIV negative men who have sex with men is calculated according to *p* = 0.42. Considering possible factors such as questionnaire or sample failure, the sample size of the HIV positive men who have sex with men group is 300, the sample size of the HIV negative men who have sex with men group is 600, and the MSW group and the HIV negative MSM group form a control in a 1:1 ratio, with a sample size of 600.

From September 2023 to April 2024, 300 HIV-positive MSM, 600 HIV-negative MSM and 600 MSW were recruited in Tianjin, China. The study participants were recruited in a stratified manner by age, with a ratio of 2:3:1 for the 18–26, 27–45, and ≥ 46 years, respectively. The questionnaire was completed by the participants in a private space after the purpose of the study was explained to them and their informed consent was obtained. The questionnaire included demographic information, knowledge related to sexually transmitted diseases, behavioral information, and willingness to receive the HPV vaccine. Individuals who had received the HPV vaccine were excluded. Education is not a limiting condition.

#### 2.2.1 Inclusion criteria for HIV-positive MSM

(1) Males aged 18 or older. (2) History of anal or oral sex with men in the past year. (3) HIV-infected individuals or AIDS patients. Recruitment of HIV-positive MSM was conducted at designated antiretroviral therapy hospital. On a voluntary basis, those who met the inclusion criteria were consecutive recruited.

#### 2.2.2 Inclusion criteria for HIV-negative MSM

(1) Males aged 18 or older. (2) History of anal or oral sex with men in the past year. (3) HIV antibody-negative. Recruitment of HIV-negative MSM was conducted in collaboration with non-governmental organizations (NGOs) in Tianjin, with strategies involving online recruitment, venue-based recruitment, and snowball sampling.

#### 2.2.3 Inclusion criteria of MSW

(1) Males aged 18 or older. (2) History of vaginal or oral sex with women in the past year. Recruitment of MSW was conducted at community health check-up institutions. On a voluntary basis, those who met the inclusion criteria were consecutive recruited.

### 2.3 Sample collection

Samples of exfoliated cells were collected from three sites for each participant: an anal swab, a genital swab, and an oral swab. Participants were instructed not to have sexual intercourse for 2 days before sampling and not to wash their genital and anal areas on the day of sampling. Three sterile cotton swabs moistened with saline were used to collect exfoliated cells from the anal, genital (glans, foreskin, urethral opening), and oral mucosa. Samples were placed in a specialized preservation solution and stored at 4°C for testing within 1 week. Additionally, a 5 mL sample of venous blood was collected for the purpose of HIV antibody testing.

### 2.4 Laboratory testing

#### 2.4.1 HPV DNA testing and genotyping

HPV DNA and genotyping were performed using the HPV23 nucleic acid typing detection kit (Kedean, Hangzhou, China), based on fluorescence PCR melting curve analysis. DNA extraction was conducted using the EB1000 nucleic acid extractor (Kedean, Hangzhou, China). High-risk HPV genotypes (16, 18, 31, 33, 35, 39, 45, 51, 52, 53, 56, 58, 59, 66, 68, 73, 82) and low-risk genotypes (6, 11, 42, 70, 81, 83) were classified according to their carcinogenicity.

#### 2.4.2 HIV serology testing

For HIV-negative MSM recruited through NGOs, initial screening was conducted using the HIV Ag/Ab colloidal gold method test kit (Zhongsheng Kejv, Tianjin, China). Samples reactive in the initial screening were confirmed with the Western blot method (MP, Singapore) and excluded from the study if positive.

### 2.5 Statistical analysis

Descriptive statistics were used for categorical variables (e.g., HPV genotype positivity, willingness to vaccinate), expressed as frequencies and percentages. Chi-square tests compared HPV genotype positivity rates and willingness to vaccinate among the three groups. Multivariate logistic regression identified factors associated with HPV infection. Adjusted odds ratios (aOR) and 95% confidence intervals (CI) were reported. Data were analyzed using SPSS 24.0 (SPSS, Inc., Chicago, IL, USA), with significance set at *p* < 0.05.

## 3 Results

### 3.1 Demographic characteristics of the participants

A total of 300 HIV-positive MSM, 600 HIV-negative MSM and 659 MSW were recruited in this study. The age distributions among the three groups were similar, with a median age of 32 (IQR, 25–40) for HIV-positive MSM, 32 (IQR, 25–39) for HIV-negative MSM, and 35 (IQR, 24–43) for MSW. With regard to marital status, the majority of HIV-positive MSM and HIV-negative MSM were unmarried (70.7 and 71.7%, respectively), while the majority of MSW were married (55.8%). In terms of education level, all three groups exhibited a high level of education, with the majority having completed college or above. With regard to occupational, all three groups were predominantly engaged in white-collar work. Socio-demographic characteristics and sexual behaviors of participants are shown in Table 1.

**Table 1:** Demographic Characteristics and sexual behaviors of the participants (*n* = 1,559).

Variables	HIV-positive MSM		HIV-negative MSM		MSW		*χ^2^*	*P*
	No.	%	No.	%	No.	%		
Age (years)							3.409	0.492
18 ~ 26	98	32.7	199	33.2	192	29.1		
27 ~ 45	152	50.7	301	50.2	340	51.6		
≥46	50	16.6	100	16.6	127	19.3		
Race							7.350	0.025
Han	283	94.3	580	96.7	644	97.7		
Minority	17	5.7	20	3.3	15	2.3		
Marriage status							235.800	<0.001
Unmarried	212	70.7	430	71.7	278	42.2		
Married	52	17.3	125	20.8	368	55.8		
Divorced /Widowed	36	12.0	45	7.5	13	2.0		
Education level							38.220	<0.001
High school and below	105	35.0	146	24.3	268	40.7		
College and above	195	65.0	454	75.7	391	59.3		
Occupation							33.651	<0.001
White collar	117	39.0	301	50.2	284	43.1		
Freelancer	108	36.0	170	28.3	157	23.8		
Others	75	25.0	129	21.5	218	33.1		
Monthly income (RMB)							112.742	<0.001
<5,000	171	57.0	252	42.0	472	71.6		
≥5,000	129	43.0	348	58.0	187	28.4		
Sexual behaviors in the last 6 months								
Homosexual intercourse	184	61.3	549	91.5	0	0	1086.010	<0.001
Consistent condoms use	93	31.0	271	45.2	401	60.8	79.445	<0.001
Heterosexual intercourse	34	11.3	106	17.7	536	81.3	673.540	<0.001
Oral intercourse	85	28.3	523	87.2	241	36.6	426.397	<0.001
STIs in the past year	71	23.7	34	5.7	8	1.2	158.215	<0.001

### 3.2 HPV infection status

The overall HPV infection prevalence was 62.0% for HIV-positive MSM, 53.7% for HIV-negative MSM, and 8.3% for MSW (*p* < 0.001). The prevalence for the genotypes covered by the 9-valent, 4-valent, and 2-valent vaccines were 47.0, 36.8, and 17.3% among HIV-positive MSM, 36.8, 25.2, and 9.5% among HIV-negative MSM, and 3.5, 2.6, and 2.1% among MSW, respectively (*p* < 0.001). The anal HPV infection prevalence for HIV-positive MSM, HIV-negative MSM, and MSW were 55.7, 42.0, and 2.1%, respectively. The genital HPV infection prevalence were 25.3, 24.7, and 6.4%, respectively. The oral HPV infection prevalence were 2.7, 3.2, and 0.6%, respectively. Co-infection prevalence at anal and genital were 20.3, 14.2, 0.6%, respectively. Co-infection prevalence at anal and genital and oral were 1.3, 0.3%, 0, respectively. Prevalence of HPV genotype and multiple HPV genotypes are shown in Table 2.

**Table 2:** The distribution of different HPV genotype infection in the participants (*n* = 1,559).

Category	HIV-positive MSM		HIV-negative MSM		MSW		*χ^2^*	*P*
	*n*	%	*n*	%	*n*	%		
Any genotype detected^I^	186^a^*	62.0	322^a^	53.7	55^b^	8.3	387.497	<0.001
Age								
18 ~ 26	56^a^	57.1	97^a^	48.7	11^b^	5.7	111.752	<0.001
27 ~ 45	93^a^	61.2	160^a^	53.2	21^b^	6.2	214.803	<0.001
≥46	37^a^	74.0	65^a^	65.0	23^b^	18.1	70.217	<0.001
Low-risk genotypes								
Any low-risk genotypes	94^a^	31.3	162^a^	27.0	20^b^	3.0	171.168	<0.001
HPV6	33	11.0	69	11.5	2	0.3	74.419	<0.001
HPV11	35	11.7	39	6.5	1	0.2	65.769	<0.001
HPV42	13	4.3	23	3.8	4	0.6	17.720	<0.001
HPV70	10	3.3	19	3.2	3	0.5	14.515	0.001
HPV81	23	7.7	44	7.3	10	1.5	28.512	<0.001
HPV83	2	0.7	6	1.0	0	0	0.016^V^	0.900
High-risk genotypes								
Any high-risk genotypes	151^a^	50.3	248^b^	41.3	37^c^	5.6	291.149	<0.001
HPV16	37	12.3	42	7.0	5	0.8	59.149	<0.001
HPV18	20	6.7	15	2.5	10	1.5	20.019	<0.001
HPV31	15	5.0	19	3.2	1	0.2	25.855	<0.001
HPV33	6	2.0	6	1.0	1	0.2	8.842	0.012
HPV35	12	4.0	19	3.2	2	0.3	18.785	<0.001
HPV39	8	2.7	10	1.7	2	0.3	10.225	0.006
HPV45	11	3.7	17	2.8	0	0	21.665	<0.001
HPV51	5	1.7	23	3.8	5	0.8	14.692	0.001
HPV52	24	8.0	44	7.3	3	0.5	44.328	<0.001
HPV53	24	8.0	31	5.2	4	0.6	36.062	<0.001
HPV56	7	2.3	18	3.0	3	0.5	12.139	0.002
HPV58	20	6.7	42	7.0	4	0.6	37.085	<0.001
HPV59	6	2.0	13	2.2	0	0	14.130	0.001
HPV66	15	5.0	30	5.0	3	0.5	26.332	<0.001
HPV68	13	4.3	26	4.3	2	0.3	24.127	<0.001
HPV73	3	1.0	7	1.2	5	0.8	0.554	0.758
HPV82	22	7.3	24	4.0	0	0	42.467	<0.001
Vaccine-preventable genotypes								
9v genotypes^II^	141^a^	47.0	221^b^	36.8	23^c^	3.5	287.133	<0.001
4v genotypes^III^	108^a^	36.0	151^b^	25.2	17^c^	2.6	195.327	<0.001
2v genotypes^IV^	52^a^	17.3	57^b^	9.5	14^c^	2.1	69.098	<0.001
Multiple genes detected								
0	114^a^	38.0	278^a^	46.3	604^b^	91.7	237.404	<0.001
1	80^a^	26.7	161^a^	26.8	42^b^	6.4	106.611	<0.001
2	59^a^	19.7	95^b^	15.8	11^c^	1.7	98.964	<0.001
≥3	47^a^	15.7	66^a^	11.0	2^b^	0.3	89.960	<0.001
low-risk and high-risk detected	59^a^	19.7	90^a^	15.0	2^b^	0.3	119.850	<0.001
Anal area								
Any genotype detected	167^a^	55.7	252^b^	42.0	14^c^	2.1	393.010	<0.001
Any low-risk genotypes	77^a^	25.7	124^a^	20.7	7^b^	1.1	153.204	<0.001
Any high-risk genotypes	135^a^	45.0	180^b^	30.0	7^c^	1.1	294.830	<0.001
Genital area								
Any genotype detected	76^a^	25.3	148^a^	24.7	42^b^	6.4	92.229	<0.001
Any low-risk genotypes	34^a^	11.3	63^a^	10.5	14^b^	2.1	43.288	<0.001
Any high-risk genotypes	54^a^	18.0	102^a^	17.0	30^b^	4.6	59.335	<0.001
Oral mucosa								
Any genotype detected	8^a^	2.7	19^a^	3.2	4^b^	0.6	11.435	0.003
Any low-risk genotypes	4^a^	1.3	6^a^	1.0	1^a^	0.2	5.315	0.070
Any high-risk genotypes	6^a,b^	2.0	15^b^	2.5	3^a^	0.5	9.183	0.010
Multi-site infections								
Co-infection at three sites	4	1.3	2	0.3	0	0	9.627	0.008
Co-infection at anal and genital	61	20.3	85	14.2	4	0.6	115.423	<0.001
Co-infection at anal and oral	5	1.7	6	1.0	0	0	9.380	0.009
Co-infection at genital and oral	4	1.3	7	1.2	1	0.2	5.780	0.056

^I^Any of the 23 HPV genotypes detected. ^II^9v genotypes include HPV 6, 11, 16, 18, 31, 33, 45, 52, and 58. ^III^4v genotypes include HPV 6, 11, 16, and 18. ^IV^2v genotype include HPV 16, and 18. ^V^Value for continuity-corrected *χ*^2^. ^*^If the letters marked are the same, it indicates that there is no difference between the corresponding two sets of data, while if the letters are different, it indicates that the difference is statistically significant (*p* < 0.05) (Same below).

### 3.3 Willingness to receive the HPV vaccine

**Figure 1:**
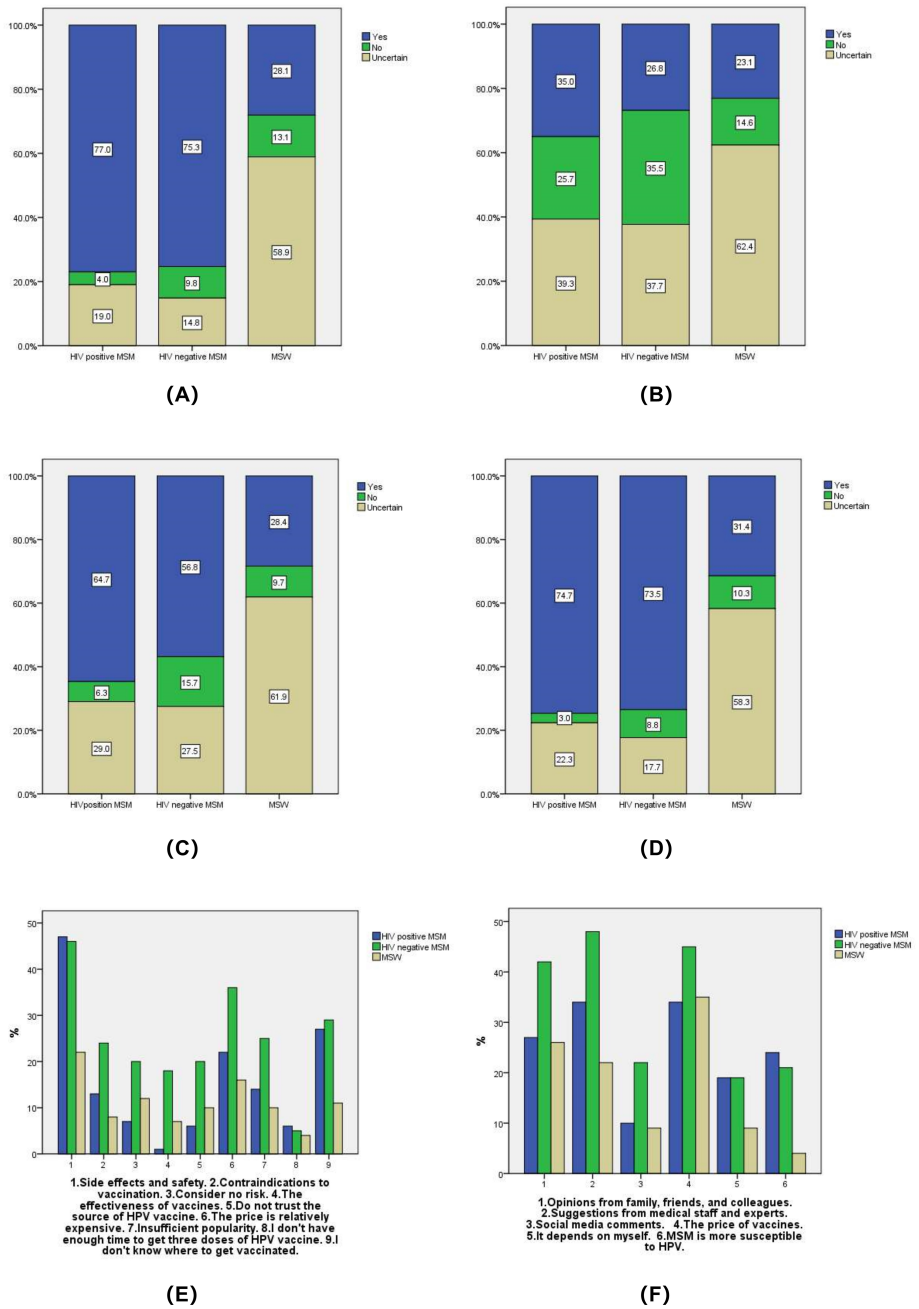
Willingness to receive HPV vaccine. **(A)** If the HPV vaccine does not restrict gender, are you willing to receive the HPV vaccine? **(B)** Do you have any family or friends who have already been received the HPV vaccine? **(C)** Would you be willing to recommend your family and friends to receive the HPV vaccine? **(D)** If the HPV test result is positive, are you willing to receive the HPV vaccine? **(E)** What is the main reason why you are unwilling to receive the HPV vaccine? **(F)** What factors will affect your willingness to receive the HPV vaccine?

Among HIV-positive MSM and HIV-negative MSM, 77.0 and 75.3%, respectively, expressed willingness to receive the HPV vaccine if it was available for men, whereas 58.9% of MSW were unwilling (*p* < 0.001). The most common reasons for unwillingness were concerns about side effects and safety, high cost, and a lack of knowledge about where to get vaccinated. The relevant issues regarding willingness to receive HPV vaccine are shown in Figure 1.

### 3.4 Factors influencing HPV infection

**Figure 2:**
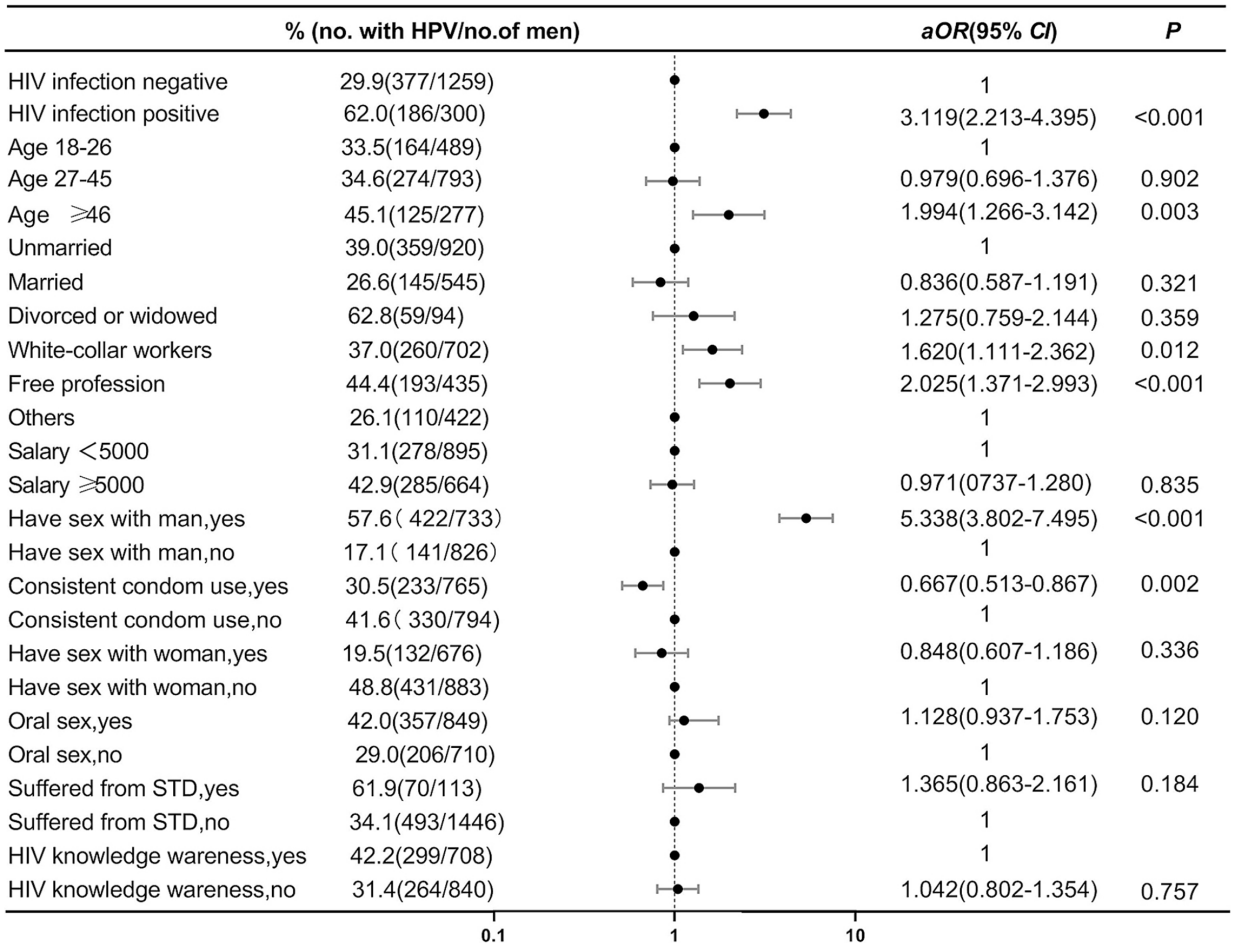
Multivariate logistic regression analysis of factors related to male HPV infection.

In the multivariable analysis, HIV-positive (aOR, 3.119; 95% CI, 2.213–4.395), over 46 years old (aOR, 1.994; 95% CI, 1.266–3.142), with an occupation of “white collar” (aOR, 1.620; 95% CI, 1.111–2.362) and “freelancer” (aOR, 2.025; 95% CI, 1.371–2.993) and having a history of homosexual behavior in the past 6 months (aOR, 5.338; 95% CI, 3.802–7.495) were risk factors for HPV infection among men in Tianjin. Consistent condoms use in the past 6 months (aOR, 0.667; 95% CI, 0.513–0.867) was found to be a protective factor for HPV infection. The results of the multivariable logistic regression analysis were presented in Figure 2.

## 4 Discussion

This study shows that the HPV infection prevalence among MSM (HIV-positive 62.0%, HIV-negative 53.7%) in Tianjin is significantly higher than among MSW (8.3%), with higher prevalence in the anal region compared to the genital and oral region. Being HIV-positive, being over 46 years old, with an occupation classified as “white collar workers” and “freelancing” and a history of homosexual behavior in the past 6 months were risk factors for HPV infection among men in Tianjin. Most MSM showed a positive willingness to receive the HPV vaccine.

The anal region is the most severely affected site for HPV infection among MSM populations. This study demonstrates that the anal HPV infection prevalence among HIV-positive MSM in Tianjin is 55.7%, which is lower than previous reports from China [65.6% (12) and 77.9% (13)], Rome (14) (83.5%), Peru (15) (77.1%), and Italy (16) (60.0%). Anal HPV infection prevalence among HIV-negative MSM was found to be 42.0%, which is also lower than previous reports from China (47.1–58.0%) (7, 11), South Africa (50.8%) (17), Rome (68.3%) (14), and Thailand (18) (59.0%), but higher than the report from Italy 15 (37.8%) (16).The reason for the difference may be that the participants in different studies have different demographic characteristics, behavioral characteristics and the course of AIDS. Other studies can be conducted in the future to verify this hypothesis.

Compared to the anal region, the genital region has a relatively lower HPV infection prevalence. This study indicates that the genital HPV infection prevalence among HIV-positive MSM is 25.3%, which is higher than that reported in Italy (18.5%). The genital HPV infection prevalence among HIV-negative MSM is 24.7%, which is higher than that reported in Taiwan (18.0%) (19), lower than that reported in Beijing (36.7%) (20), and comparable to the report from Italy (23.5%) (15). Among the three surveyed sites, the oral region has the lowest HPV infection prevalence. The oral HPV infection prevalence among HIV-positive and HIV-negative MSM in Tianjin are 2.7 and 3.2%, respectively, which is similar to the report from southern China (2.7%) (7). The data from Tianjin, situated in northern China, combined with data from southern China, suggests that the oral region is currently not a high-risk site for HPV infection among MSM.

There is a significant difference in the prevalence of HPV infection between MSM and MSW. The overall HPV infection prevalence among MSW is 8.3%, which is similar to the prevalence reported in Guangxi (21) (10.5%) and Jiangsu (22) (11.31%) in China. The genital HPV infection prevalence among MSW is higher than that in the anal region (6.4 vs. 2.1%), a trend also reported in Guangxi (10.8 vs. 3.8%) (21). However, international reports indicate that HPV infection prevalence among MSW was considerably higher, with a global overall prevalence of 50.8% (23) and a study from the United States reporting a genital HPV infection prevalence of 45.2% (95% CI, 41.3–49.3%) among men aged 18–59 (24), indicating significant regional differences worldwide.

The HPV infection prevalence for the genotypes covered by the 9-valent, 4-valent, and 2-valent vaccines are higher in HIV-positive MSM compared to HIV-negative MSM and MSW. The HPV infection prevalence for the genotypes covered by the 9-valent and 2-valent vaccines in HIV-positive MSM is lower than that reported from South Africa (47.0 vs. 75.0%, 17.3 vs. 20.0%), while the 4-valent rate is higher (36.0 vs. 35.0%) (17). In HIV-negative MSM, the rates for the 9-valent and 4-valent genotypes are higher than in South Africa (36.8 vs. 28.0%, 25.2 vs. 13.0%), but the 2-valent rate is lower (9.5 vs. 10.0%) (17). The HPV infection prevalence for the genotypes covered by the 9-valent, 4-valent, and 2-valent vaccines in MSW are lower than the rates reported in a global meta-analysis (3.5 vs. 16.0%, 2.6 vs. 11.0%, 2.1 vs. 7.0%) (25).

HPV vaccination can effectively reduce the prevalence and mortality of related diseases among MSM in China, particularly anal-genital warts with the 4-valent vaccine and anal cancer with the 9-valent vaccine (26). Our study found that over two-thirds of MSM would be willing to get vaccinated if the HPV vaccine were available to men, which is slightly lower than that reported in other studies from China (87.6%) (27). However, most MSW are reluctance to get vaccinated, mainly due to concerns about vaccine side effects and safety, vaccine cost, and lack of knowledge about vaccination locations. Studies conducted in Quebec, France, and other regions have also found that safety concerns are a significant barrier to HPV vaccine acceptance (28, 29). Factors influencing HPV vaccine uptake among men in China include vaccine cost, recommendations from healthcare providers and experts, and opinions from family and friends. Other studies have also shown a positive correlation between the advice of significant others and the HPV vaccination rate (30). To further enhance vaccination willingness and improve cost-effectiveness, it is necessary to adjust vaccine prices appropriately or reduce the economic burden of vaccination through other means. Research has demonstrated that individuals who receive HPV vaccine recommendations from healthcare providers are more likely to be vaccinated (31). To enhance vaccination rates, it is essential to implement strategies that strengthen publicity and education through various channels such as hospitals, communities, and online platforms. Additionally, government regulation of vaccine prices, enhanced training for healthcare workers, and tailored interventions based on different population needs, can also be employed to enhance vaccination rates.

Our multivariate analysis indicate that being HIV-positive, aged 46 or above, and a history of same-sex behavior in the past 6 months are risk factors for HPV infection among men in Tianjin. The specific sexual behaviors of MSM, particularly anal intercourse, increase the risk of HPV infection due to mucosal damage. It has been demonstrated that HIV infection can induce immunosuppression, thereby increasing the risk of HPV infection and prolonging the persistence and reactivation of latent HPV. Consequently, HIV-positive MSM are a priority group for anal cancer screening research (32). Our study observed a higher risk of HPV infection among older MSM, which contrasts with some previous studies indicating that the prevalence of anal HPV infection among MSM does not vary with age (33, 34). It is important to note that the infection of HPV in middle-aged and older men, as older MSM may have higher cumulative HPV infection, warranting increased HPV screening for middle-aged and older MSM. Since there is no specific HPV vaccination plan for men in Chinese Mainland at present, and the vaccines are approval in other countries up to 45 years old, so vaccination is not relevant for the oldest group in the study, and cancer screening is the only practical recommendation. However, our findings also indicate that nearly half of MSM younger than 27 years old were already infected with HPV. This highlights the importance of vaccinating before sexual debut for better prevention, which is also a long-term solution for preventing anal cancer in MSM (35).

Interestingly, we found that white-collar workers and freelancers are risk factors of HPV infection compared to other professions. It is worth noting that the “other” category in this study includes some university students who have been in society for a shorter time and have a higher level of education, which may contribute to their better awareness of disease transmission and safe sexual practices, possibly explaining the lower HPV infection rate in this group.

This study strictly followed the inclusion criteria for subjects, recruiting participants through a stratified snowball sampling method both online and offline, which yield a good representativeness sample. However, this study is subject to certain limitations. Firstly, data collection is based on self-reporting, which may be subject to recall bias and potentially incomplete, truthful responses due to the personal nature of sexual behavior. Secondly, this study was conducted in Tianjin, China, and the HPV infection prevalence assessment may not represent the national level, therefore caution is needed when extrapolating the results. Thirdly, this study is a cross-sectional survey and cannot confirm the causal relationship between HPV infection and its influencing factors.

Overall, the findings of this study indicate that the prevalence of HPV infection among MSM in Tianjin is significantly higher than among MSW, with the anal region having a higher infection rate than the genital region. HPV infection among men is associated with HIV infection, age 46 or above, and same-sex sexual behavior. There is a need to promote HPV prevention and intervention measures for this population, particularly focusing on older MSM, and to include anal cancer screening in the follow-up and treatment of HIV-positive individuals. The high prevalence of HPV genotypes covered by vaccines among MSM underscores the necessity for HPV vaccination programs tailored to this group. Further research and technical preparation for such vaccination initiatives are essential to address this public health concern effectively.

## Data Availability

The raw data supporting the conclusions of this article will be made available by the authors, without undue reservation.
